# Advancing Semen Evaluation Using Lipidomics

**DOI:** 10.3389/fvets.2021.601794

**Published:** 2021-04-16

**Authors:** Holly C. Evans, Thu T. N. Dinh, Madison L. Hardcastle, Alicia A. Gilmore, Muhammet R. Ugur, Mustafa Hitit, Frank Dean Jousan, Molly C. Nicodemus, Erdogan Memili

**Affiliations:** ^1^Department of Animal and Dairy Sciences, Mississippi State University, Starkville, MS, United States; ^2^Department of Animal Genetics, Kastamonu University, Kastamonu, Turkey

**Keywords:** fatty acids, lipids, biomarkers, livestock, cryotolerance

## Abstract

Developing a deeper understanding of biological components of sperm is essential to improving cryopreservation techniques and reproductive technologies. To fully ascertain the functional determinants of fertility, lipidomic methods have come to the forefront. Lipidomics is the study of the lipid profile (lipidome) within a cell, tissue, or organism and provides a quantitative analysis of the lipid content in that sample. Sperm cells are composed of various lipids, each with their unique contribution to the overall function of the cell. Lipidomics has already been used to find new and exciting information regarding the fatty acid content of sperm cells from different species. While the applications of lipidomics are rapidly evolving, gaps in the knowledge base remain unresolved. Current limitations of lipidomics studies include the number of available samples to analyze and the total amount of cells within those samples needed to detect changes in the lipid profiles across different subjects. The information obtained through lipidomics research is essential to systems and cellular biology. This review provides a concise analysis of the most recent developments in lipidomic research. This scientific resource is important because these developments can be used to not only combat the reproductive challenges faced when using cryopreserved semen and artificial reproductive technologies in livestock such as cattle, but also other mammals, such as humans or endangered species.

## Introduction

### Fertility—An Economically Vital Trait

Male fertility may be defined as the ability of a sperm cell to fertilize an oocyte and support development to produce viable offspring. There are numerous factors that contribute to the overall fertility of a given male. The variation within these factors makes it challenging to determine what makes one male more fertile than another. Previous research in humans has demonstrated that aspects of the ejaculate, such as sperm volume, presence of abnormal components (i.e., urine or blood), and seminal plasma volume, along with more specific spermatozoan characteristics including morphology, motility, DNA integrity, acrosome integrity, and membrane integrity can further illustrate differences of fertility and performance (1, 2). However, these characteristics alone may not be enough to ascertain the true fertility of an individual male. With technological advancements in animal management toward highly efficient and productive livestock, this industry has shifted toward the utilization of sperm cryopreservation techniques to allow for the broader distribution of top-quality genetics, while preserving their impact for future generations.

Cryopreservation is the freezing of biological samples with the intent of preserving the integrity of the sample for later use (3). In the case of spermatozoa, the frozen sample would later be used for artificial insemination (AI) of an open female. Freezing and thawing semen samples can be extremely detrimental to sperm due to cellular damage, membrane breakage, acrosome damage, and cell death that is caused by ice crystal formation, reactive oxygen species (ROS), lipid peroxidation, and other factors (4). However, an individual male that maintains and produces high-quality, fresh sperm could perform at critically lower levels after cryopreservation due to damages incurred during the process of cryopreservation. Cryopreserved sperm still suffer from structural damage that affect sperm physiology including low motility and viability that results in the considerable extent of fertility loss (5, 6), and yet, advances in knowledge concerning semen cryopreservation protocols have led to the commercialization of this process to market genetically superior livestock on a worldwide basis with tremendous positive economic impact.

### Spermatogenesis

Spermatogenesis is the continuous generation of sperm cells in the male, which ensures the replenishment of spermatozoa. In bulls for instance, the process of spermatogenesis takes ~61 days to complete with new cells being added to the process every 13.5 days (7)). Spermatozoa, along with various hormones, proteins, and seminal fluids, are produced in the testicles, which contribute to normal reproductive physiological processes in males. Testicles are the site of testosterone production. Inhibin is produced by Sertoli cells within the testicles and elicits negative feedback on sperm production and estrogen release, aiding in spermatogenesis, the modulation of male libido, and the male erection (8).

Spermatogenesis is compartmentalized in the seminiferous tubules of the testicles. In the basal compartment, mitosis takes place to allow for the proliferation of spermatogonia to obtain the primary spermatocytes. Primary spermatocytes undergo two cycles of meiosis to further mature to become secondary spermatocytes, and then, spermatids. Spermiogenesis, the final stage of spermatogenesis, completes the cellular transformation of spermatids into mature, motile spermatozoa. Finally, spermiation allows for mature spermatozoa to be released from the Sertoli, or sustentacular, cells and into the lumen of the seminiferous tubules. Spermatozoa are stored in the caput (head) of the epididymis. While in the caput of the epididymis, sperm are immotile and have a low membrane fluidity due to the high cholesterol to phospholipid ratio (9). When sperm reach maturity, they are pushed into the corpus (body) of the epididymis. On a molecular basis, mature sperm will have a higher degree of membrane fluidity, contain disulfide bonds, and have lower cholesterol to phospholipid ratio than immature sperm cells (9). From the corpus of the epididymis, mature spermatozoa are moved into the caudal (tail) of the epididymis for storage and transport to the vas deferens to await ejaculation. Sperm are altered as they move through the epididymis, including the modification of lipids and sugars found on the sperm cell's plasma membrane (10). This allows for the development of cellular motility and changes in membrane fluidity in preparation for fertilization.

### Sperm Structure

Sperm cells have several structures that allow for the fertilization of the female oocyte. The head piece of the bovine sperm cell is shaped like a round kernel of corn. In other models such as the rooster, the sperm head is slender and elongated (11). If the head of the sperm cell, regardless of species, is not properly shaped, its ability to maneuver with forwarding, progressive motility to fertilize an oocyte can be compromised. Abnormalities have been associated with immaturity of sperm and reduced fertility (12).

The sperm head is encapsulated by several membrane layers which include theplasma membrane, the outer acrosomal membrane, the acrosome, and the inner acrosomal membrane. The acrosome, which deteriorates once the acrosome reaction occurs, allows for penetration of the zona pellucida (13). Through capacitation, the acrosomal matrix proteins are exposed and allow for interaction with the zona pellucida of the oocyte (14). Proteins such as sp56 and zonadhesin have been identified as key sperm-zona binding agents (15, 16). The nucleus of sperm cells is held within these layers to prevent damage to the genetic material needed to fertilize an oocyte for successful fetal development. On the front portion of the sperm cell, the acrosome bends posteriorly to create the apical ridge, which is responsible for the recognition and binding of the cell to the zona pellucida of the oocyte (17). Toward the center of the sperm head, the acrosome ends and forms the equatorial segment. The structures of the head that lie between the apical segment and the equatorial segment are grouped into the principal segment of the sperm head.

The midpiece (neck) of the spermatozoa connects to the implantation socket at the caudal end of the sperm head via the capitulum. Mitochondrial adenosine triphosphate (ATP) production occurs in the midpiece and fuels the movement of sperm cells. The midpiece is composed of mitochondrial helices and inner tubules that are responsible for the bending of the sperm tail. There are coarse outer fibers that form a fibrous sheath surrounding the sperm tail. The endpoint of the midpiece is the annulus. The axial filament complex originating from the distal centriole is a matrix of the microtubules. The sliding motion of these tubules allows for the lateral movement of the sperm tail and is driven by ATPases (18). This is of great importance because ATP production is dependent upon environmental temperature, which allows for greater mobility. Mitochondria are biomarkers of sperm cell fertility and are necessary for motility (19). They have two sets of membranes, the inner and outer mitochondrial membranes. This creates an environment for energy-transduction and allows for oxidative phosphorylation to occur (20).

## Lipids of the Sperm Membrane

### Lipid Classes

Lipids are biological components that are broadly defined as naturally occurring substances that are not soluble in water. From storing energy to providing structure or flexibility to cellular components, lipids have variable functions in a biological system. There are many ways to classify lipids which range from simple to complex categorization. For this discussion, the Lipid Metabolites and Pathways Strategy (LIPID MAPS®) classification system will be utilized (LIPID Metabolites and Pathways Strategy; http://www.lipidmaps.org). LIPID MAPS classify lipids into eight different categories: fatty acyls (FAc), glycerolipids (GL), glycerophospholipids (GP), sphingolipids (SP), sterol lipids (ST), prenol lipids (PR), saccharolipids (SL), and polyketides (PK) (21). Of the listed lipid classes, they can be further separated based on their basic subunits. Ketoacyl subunits are found in polyketides, saccharolipids, fatty acyls, glycerolipids, glycerophospholipids, and sphingolipids (22). Ketoacyl subunits are acetyl compounds and propionyl compounds (21). The remaining lipid classes of sterols and prenol lipids contain organic isoprene subunits, which are unsaturated hydrocarbon compounds.

Lipids classified within the fatty acyl (FAc) group are synthesized via chain elongation by acetyl-CoA with malyonyl-CoA. The FAc group is composed of diverse lipids that are further classified into subgroups. The FAc group can be thought of as one of the most fundamental building blocks in biological lipids because they are the foothold, if you will, for the formation of larger, more complex lipids. Glycerolipids typically function as an energy storage system in mammalian tissue. Mono-, di-, or tri-substituted glycerols comprise glycerolipids (GL). However, GLs are also play a major role in both cell signaling and act as structural components of cell membranes. These molecules are composed of neutral lipids and have a glycerol backbone (23). One of the most widely recognized GLs are triglycerides, which store energy in the body in the form of glycerol unit and three long-chain fatty acid units. These units are broken down via β-oxidation to help fuel the body with energy when sugars are not available or when exercise is prolonged.

Glycerophospholipids (GP), or phospholipids, are long-chain fatty acids that perform as both structural and functional components of cells. In the most basic of forms, GPs are composed of a negatively charged phosphate head, two fatty acids, which can vary, and a glycerol molecule. They are readily observed in the membranes of cells, including lipid bilayers, in addition to serving as secondary messengers and binding sites. This is because the fatty acid chains, or tails, are uncharged and hydrophobic, whereas the phosphate group is hydrophilic. These lipids assist with cellular signaling and metabolism in both animal and plant cells (24, 25). Lipids with a backbone of sphingoid bases are grouped into the SP category. These lipids also contain aliphatic amino alcohols like sphingosine, which is an important phospholipid. These lipids are pivotal to the vitality and protection of cellular membrane protection. This is due in part to the formation of outer leaflets of the plasma membrane lipid that are not only stable structurally, but also resistant to chemical changes (26).

Cholesterol and its derivatives are components of the sterol lipids (ST) category. Cholesterol is commonly discussed in human medicine due to its role in cardiovascular disease development and control. This wax-like lipid is necessary for normal bodily functions, but it can become harmful in copious amounts. On a cellular level, cholesterol and other ST lipids give structure to membranes. Cholesterol efflux in the plasma membrane of sperm can alter acrosomal responsiveness, and thus, negatively impact fertilization success due to changes in stability (27, 28). In rodent models, high-fat diets decreased sperm motility, increased serum lipid concentrations, and altered hormone levels to include heightened estradiol levels and decreased testosterone levels (29). In addition to structural functions, sterol lipids also act as signaling molecules and hormones. General groups of these include progesterone, estrogen, and androgen.

Prenol lipids include classes of vital compounds such as vitamins K and E, which act as antioxidant agents, preventing cell membrane oxidation and controlling the production of free radicals. They are synthesized from 5-carbon precursors, which include compounds like isopentenyl diphosphate and dimethylallyl diphosphate (30). These products are a result of the mevalonic acid pathway (30). Additional functions include cell signaling and anabolism.

Lipids that have a sugar backbone where fatty acids attach are grouped into the saccharolipids (SL) category. Saccharolipids have a similar structure to that of glycerolipids because the glycerol is replaced with monosaccharides. The structure of SLs is compatible with lipid bilayers. Polyketides are considered to be secondary metabolites. They are synthesized by the polymerization of acetyl and propionyl subunits. This is made possible by classic, iterative, and multimodular enzymes, which share features from a mechanical standpoint with fatty acid synthases. This group of molecules may contain methylene groups or varying carbonyl groups. Polyketides are important from a pharmaceutical standpoint in that PKs are often components of anticancer, antifungal, anticholesteremic agents, antibiotics, immunomodulators, and parasiticides due to their antimicrobial and immunosuppressive qualities (21). Within the simple category are STs, such as cholesterol and FAc (including all derivatives), compared to the complex category, which would include SPs, GLs, and GPs (31). While new technology is being applied to the study of lipidomics, commonly used tools include, but are not limited to, GC, MS, LC-MS, thin layer chromatography, and NMR.

As FAs are structural compounds of cell membranes, the composition of the fatty acids may play a critical role in sperm function through regulation of membrane structure (32). Dietz et al. (33) suggested lipid concentration of bovine semen to be 4.10 mg/ml and were able to identify a total of nine fatty acids: SFA 12:0, 14:0, 15:0, 16:0, 16:1, 17:0, 18:0, MUFA 18:1, and PUFA 18:2. Of those, 16:0 (palmitic acid) was the most abundant lipid group with a relative percentage of 40.9% followed by palmitic acid 14:0, 18:0, and 18:1 as the most predominant FA with relative concentrations of 26.4, 12.9, and 10.5%, respectively. Komarek et al. (34) analyzed the lipid composition of bull sperm and seminal plasma samples separately using thin-layer chromatography and reported that total lipid content of bovine spermatozoa and seminal plasma accounts for 12.0 and 1.35% of the total dry weight, respectively. Fractions of lipids were detected, including phospholipids, cholesterol, diglycerides, triglycerides, and wax esters (34). The most abundant lipid groups were phospholipids and cholesterol with 73 and 14.5% of the total lipid composition, respectively.

### Cholesterol and Precursors of Steroid Hormones

Cholesterol is a steroid hormone found in all mammals. Cholesterol serves as the precursor molecule for all other steroid hormones, as well as, to vitamin D and bile acids/salts. There are five major classes of sterol hormones: androgens, estrogens, progestogens, glucocorticoids, and mineralocorticoids. Androgens are especially important in males due to their role in fertility and reproduction. Testosterone is a cholesterol derivative responsible for the development of the male's sexual behavior, maintenance of the testes, the onset of puberty, and development of muscle mass (35). While cholesterol serves as a building block for sterol hormones, cholesterol also performs an important structural function in cellular membranes and contributes to the fluidity of plasma membranes and their functionality (36). Cholesterol is key for the process of capacitation of sperm cells. Amounts of the cholesterol in sperm membranes may determine cryotolerance of the cell because higher levels of cholesterol result in more rigid and cohesive sperm membranes. Bull sperm (0.89 μM/10^9^ sperm) and ram sperm (0.722 μM/10^9^ sperm) contain lower levels of cholesterol compared to human sperm (1.438 μM/10^9^ sperm) (37). In addition, the ratio of the polyunsaturated FAs to saturated FAs in bull sperm (3.5) are greater than human (1.0) and ram sperm (2.5) (38). Researchers have also studied comparative cholesterol content in neutral lipids of sperm and seminal plasma from bulls and water buffalos. While the cholesterol content of the sperm cells and seminal plasma from the bull were 23.3 and 18.8%, respectively, these values in water buffalo were 22.2 and 24.7%, respectively (39).

The loss of cholesterol from the sperm membrane leads to an imbalance that affects its permeability (40). This membrane alteration allows for calcium, bicarbonate, and potassium ions to cross freely through the membrane, thus, increasing the internal ion concentration. As the intracellular ion concentration increases, the acrosome reaction is induced. A method has been developed for total lipid extraction and purification that is still widely used with modifications for cholesterol analysis (41). The method developed employs methanol and chloroform as analytical reagents. When using the Bligh and Dyer method, the volumes of cholorform:methanol: water, both before and after dilution, should be kept in the proportions of 3:2:0.8 and 2:2:1.8, respectively. The ratios presented account for water present within a given sample. For samples with higher water volume, methanol, and chloroform volumes should be adjusted. Samples lacking water volumes can de diluted with water. Samples are prepared using a vortex and centrifugation to establish distinct layers, a chloroform layer and an organic layer which contains the lipids. The organic layer containing the lipids is then separated and evaporated under liquid nitrogen. Samples can then be analyzed using the preferred method of the researcher, such as microscopy or LC-MS (42, 43). Previous studies have focused on manipulating cholesterol levels to determine the effect on post-thaw viability (44). In a rodent based study, it was determined that rabbits that were fed high-fat diets had significantly lower semen quality, motility, capacitation, and acrosome reaction (45). This could be a result of increased cholesterol incorporation to the plasma membrane, which increases membrane rigidity and resistant to alteration by reducing the fluidity. The ability to quantify cholesterol within the sperm membrane allows for the ratio comparison of cholesterol to other lipids as well as proteins in both high and low freezability and fertility sperm, allowing for a clearer picture of the dynamics.

### Fat-Soluble Vitamins

Lipids are also transporters of vitamins A, D, E, and K, which contribute to functions and metabolism in the body. Vitamin E, which is found in the cell membrane, has been demonstrated to have important antioxidant properties. It destroys free hydroxyl radicals and superoxide anion, reducing lipid peroxidation of the plasma membrane (46). In study completed by Hu et al. (47) vitamin E was used as a supplementation at various concentrations to bull sperm subjected to cryopreservation. When samples were supplemented with 1.5 mg/ml concentrations of vitamin E, there was a significantly improved level in sperm motility, straight-line velocity, and straightness (*P* < 0.05). In addition, the percentage of acrosome-intact and membrane-intact sperm was significantly improved (*P* < 0.05). While vitamin E supplementation has demonstrated the ability to reduce the potential of lipid peroxidation, allowing for improved semen quality post-thaw, this is still an area of research interest to further evaluate the role of vitamin E in reproduction.

Vitamin A is required for normal mammalian spermatogenesis and has antioxidant properties. This vitamin breaks chains by attaching to peroxyl radicals, thus preventing lipid peroxidation (48). Zervos et al. (49) examined the effects of vitamin A on acrosin activity. Fifteen rams were split into three groups and received different concentrations of vitamin A, given as retinyl acetate. The three groups included a control group, the 12,500 IU/animal per day group, and the 50,000 IU/animal per day. Acrosin activity was measured using spectrophotometry. There was no statistical difference found between the control group and 12,500 IU group, but a significant decrease in acrosin activity was found in the 50,000 IU groups in comparison to the control group (*P* < 0.05). It was concluded that excessive vitamin A intake does not affect acrosin activity, but deprivation of vitamin A can reduce acrosin activity.

Vitamin D is thought to function in regulating intracellular Ca and Ca-binding proteins in the testis. Jueraitetibaike et al. (50) investigated the associations between seminal plasma vitamin D levels and semen quality. Vitamin D levels were detected using electrochemiluminescence in 220 fertile men. Seminal plasma 25(OH)D levels were positively correlated with semen volume and kinetic values of the sperm cells. Research suggests that vitamin D in seminal plasma could be linked to the regulation of sperm motility by promoting ATP synthesis via the cAMP/PKA pathway.

Vitamin K is a key modulator of extracellular calcium homeostasis within sperm cells and the epididymis, facilitates energy production within the mitochondria, and contains antioxidant properties. The intracellular compartmentalization of the vitamin K cycle may contain a more localized defense system against ROS attack (51). In addition, the reduced form of vitamin K, KH2, has been demonstrated to protect plasma membranes from peroxidation by ROS uptake in humans (52).

### Oxidation of Membrane Lipids—Primary Oxidation, Secondary Oxidation

Sperm cells are highly susceptible to oxidative stress (OS) due to the concentration of PUFAs found within the plasma membranes. Antioxidant concentrations are low in the cytoplasm of sperm cells as compared to that of somatic cells, which have larger quantities that contribute to defending against oxidative damage. Oxidative stress is the imbalance between reactive oxidative species (ROS) and antioxidants (4). Several types of ROS exist, including oxygen free radicals, non-radical species, and reactive nitrogen species. Oxygen free radicals are highly reactive compounds that can affect any cellular component (53). Examples of oxygen free radicals include compounds such as hydroxyl radicals and superoxide anions. Non-radical species are moderately reactive and are formed after both protonation and univalent reduction occur. Some examples of these would include hydrogen peroxide and hypochlorous radicals (54). These compounds react with proteins and form other ROS-like hydroxyl radicals.

Both the oxygen free radicals and the non-radical species are created by the partial reduction of oxygen within a given compound (55). Reactive nitrogen species are a little different from the other two categories because they are produced by enzymatic activity of nitric oxide synthase 2 and NADPH oxidase. Additionally, these compounds are derived from nitric oxide compounds (56). Two examples of these antimicrobial molecules include superoxide and nitric oxide. There are several potential consequences to an overabundance of ROS. One major consequence of OS is lipid peroxidation, which compromises the integrity of cell membranes (57). However, many laboratory techniques have been developed to measure lipid peroxidation in spermatozoa and to combat ROS from harming spermatozoa (57, 58).

Sperm lipids are abundant in the membranes and they are largely in the form of PUFAs, which contain unconjugated double bonds between methylene bridges (59). The double bond adjacent to methylene group weakens the methyl carbon–hydrogen bond, thus, making hydrogen excessively vulnerable to oxidative damage. Because the intracellular levels of ROS elevate excessively, ROS establishes a cascade of reactions, which eventually culminate in lipid peroxidation (LPO) (60–62). Then, a great amount of membrane fatty acids is demolished, and fluidity decreases with the loss of function of sperm cell (63). The functions of membrane receptors and enzymes are suppressed (64). Therefore, LPO initiates an autocatalytic self-propagating chemical reaction, which causes unsuccessful fertilization due to impairment of sperm function (59, 60, 65).

The machinery of lipid peroxidation can occur in three main stages: initiation, propagation, and termination. Initiation mainly comprises abstraction of hydrogen from the carbon–carbon double bonds, therefore, leading to free radicals, which then, produces lipid radicals, and subsequently, interacts with oxygen, generating the peroxyl radicals (60, 66). The chain of autocatalytic reactions is preceded with abstraction of hydrogen atoms from the PUFA by peroxyl radicals, leading to formation of organic hydroperoxides, one of the possible limiting factors of the lifespan of mammalian sperm (67). With interaction of the formed radicals with successive lipids, the propagation stage progresses with the formed radicals that then produce cytotoxic aldehydes due to decay of hydroperoxide (68, 69). Subsequently, the development of alkyl and peroxyl radicals maintained in a repeated cycle until the end product is produced as malondialdehyde (MDA) and 4-hydroxynonenal (HNE), and the chain reaction ceases. The physiological levels of lipid peroxidation indicate the functional effects of ROS on sperm metabolism improving the ability of sperm to contact with oocyte (70). Nevertheless, the lipid peroxidation is regarded as the primary molecular mechanism (71) implicated in the oxidative damage to the cell that induces death. The two major consequences of this are structural damage to cell membrane and production of secondary products (72).

PUFAs with the presence of double bonds are susceptible to free radical attack and induction of LPO, which results in morphological and membrane abnormalities, in addition to impaired motility (57, 73). In this regard, due to free radical attack on PUFA in sperm, the lipid peroxidation cascade through mitochondrial generation of ROS propels cytotoxic lipid aldehydes such as 4-hydroxynonenal (4HNE) (74). Hence, mammalian sperm has been reported to be susceptible to loss of motility (75, 76) and acrosome integrity (77) due to the exogenous oxidant as a result of LPO. This may arise from the set of complexes of acrosome reaction which causes changes in membrane phospholipid/cholesterol ratio, membrane fluidity, and net charge of sperm cellular surface because the lipid composition and metabolism play a significant role in mammalian acrosome reaction (78).

Moreover, excessive production of ROS in cryopreservation causes alterations in the levels of carbohydrate, protein, and lipid in the sperm membrane, owing to the reduction of disulfide bonds between membrane proteins (79) and the increase in the peroxidation of membrane phospholipids, along with changes of sperm glycocalyx. As a result of peroxidative damage, phosphatidylcholine, phosphatidylethanolamine, and cholesterol molecules are released along with loss of phosphatidylcholine and phosphatidylethanolamine (67, 80). This leads to ultrastructural alterations of sperm plasma membrane in which cryopreservation influences membrane integrity severely (6, 81).

Although fresh sperm had slight lipid peroxidation, cryopreserved sperm suffer from higher lipid peroxidation (82, 83). This may result from the reason that cryopreserved sperm cells can be more susceptible to peroxidases than fresh sperm cells (84) and endogenous phosphatidylcholine is subject to excessive peroxidation, which is detected particularly in the mitochondrial midpiece and tail (85). Ram sperm, due to its high sensitivity to lipid peroxidation, demonstrated greater vulnerability to chromatin damage (86), owing to changes in expression of genes regulating the protamination process, and in bulls, it is sperm positively correlated with DNA integrity (87, 88). Also, this is consistent with results that cryopreserved bovine sperm suffered from low chromatin damage when low levels of lipid peroxidation were experienced (89).

### Roles of Lipid Components in Cryopreservation

Cryopreservation and the shipment of frozen semen are necessary for the advancement of the livestock industry as it allows for customized breeding of females to genetically superior sires, thereby, increasing the progeny from these males. Nevertheless, sperm from certain sires are more resilient to cryopreservation than others due to differences among lipid compositions of the sperm cell membranes (72, 90). There are differences in the composition of spermatozoa within an ejaculate, in addition to the quantity and quality of components among sperm cells, but the ability of the sperm cell to migrate through the reproductive tract of the female to fertilize an oocyte is dependent upon the form and function of the anatomical piece being evaluated.

The protein to phospholipid components, as well as the ratios of proteins to phospholipids and cholesterol to phospholipids, vary greatly when comparing the component constituents of the plasma membrane to the outer acrosomal membrane (91). The protein to phospholipid ratio is the greatest in whole sperm, followed by the outer acrosomal membrane and then the plasma membrane, due in part to their form and function (91). The cholesterol to phospholipid ratio is lower in the whole sperm and the outer acrosomal membrane, but the plasma membrane has a greater ratio of those components (91). While much is already understood with regards to their responsibilities and functions as energy sources and structural components to cells, the role of fatty acids in fertility and cryopreservation has not been well-elucidated (92). Docosahexaenoic acid (DHA, 22:6), has been positively correlated with sperm motility and improved semen parameters under heat-stress conditions, but the mechanism of how DHA affects motility is not well-understood (92, 93). Additionally, DHA and stearic acid (18:0) are involved in motility parameters before and after freezing sperm and having high quantities of these fatty acids generally means that sperm will have better post-cryopreservation motility than those with lower quantities (94). In a study performed by Maldjian et al. (95), the introduction of 3% fish oil to the diet of boars increased DHA content in sperm from 33 to 45% and increased ejaculate concentrations but did not improve or preserve sperm parameters upon post-thaw.

Fertility and functionality of sperm cells are impacted by the structural characteristics of the spermatozoon itself. Membrane layers surrounding the nucleus and cytoplasm, as well as the tail, all contain critical lipids and fatty acids that are vital to cellular integrity and overall functionality (96). For example, the head and tail of bull sperm contain large quantities of very long-chain fatty acids followed by saturated fatty acids with choline being a predominant portion in both the head and the tail (97). Saturated fatty acids (SFA), monounsaturated fatty acids (MUFA), and polyunsaturated fatty acids (PUFA) make up the composition of lipid membranes in addition to other materials such as sugars and proteins. These components are vital to successful fertilization. This membrane matrix varies from male to male and from cell to cell within an ejaculate. Compositional characteristics of the plasma membrane give way to fluidity and freezability of sperm cells, and sperm cells with more fluid membranes display improved responses after cryopreservation procedures (98). Destabilization of the membrane is caused by temperature-induced stress in addition to osmotic stressors like water or cryoprotectants, causing damage or swelling of the membrane (99). However, detailed mechanisms behind the functionality of fatty acids in these fluid membrane roles are not well understood. Sperm with greater proportions of PUFA compared to SFA tend to demonstrate higher fertility due to the degree of fluidity and strength of the cell membrane that is provided by PUFA having multiple double bonds (94). Saturated fatty acids do not contain double bonds and are less structurally stable when encountered by stressors or challenges such as freezing temperatures from cryopreservation.

The plasma membrane of the sperm cell can be destroyed by osmotic stressors, ice crystal formation, and dehydration of the membrane from cooling rates (100, 101). These factors disrupt the integrity of the cell and hinder the ability of the membrane to be selectively permeable to important molecules, leaving them incapable of delivering genetic material to the oocyte and prevents pregnancy. In addition to this damage, sperm cells with smaller acrosomes could be at greater risk for damage or attack by these factors (102). Specie differences exist in lipid compositions of the sperm plasma membrane, as well as variations among sires within a given species, thereby, making lipid profiling a vital component to sperm evaluation.

### Lipidomics—Study of Lipid Composition and Functions

Lipidomics is the study of the lipid profile (lipidome) within a cell, tissue, or organism and provides a quantitative analysis of the lipid content in the sample being studied. This can also be thought of as a branch of metabolomics, which is the characterization and quantification of the major classes of metabolites in a given sample. Lipidomics has already been used to find new and exciting information regarding the fatty acid content of sperm cells from different species. In the stallion, mass spectrometry revealed the presence of (O-acyl)-ω-hydroxy-fatty acids, specifically in the sperm head and tail, which had not been previously detected (103). While the exact functions of these compounds are unclear, complex fatty acids, such as (O-acyl)-ω-hydroxy-fatty acids, which contain carbon chains of up to 52 carbons, are important to sperm cell membrane functionality (103). In canine species, changes in the fatty acid composition of sperm cells throughout the process of sperm maturation have been documented. The concentrations of SFA, MUFA, and PUFA were high in those sperm cells that were collected from the cauda epididymis. In addition, sperm collected from the cauda portion of the epididymis had significantly greater amounts of 8:0, 18:0, and 15:0 as compared to that found in sperm from the caput and corpus of the epididymis. Differences were also present in the epididymal fluids of samples, with the caput having significantly less 18:0, 15:0, and 18:2 than that of the cauda fluid (104). In boars, the supplementation of both n-3 and n-6 fatty acids to the diet was shown to alter the composition of sperm cell fatty acids and had a positive correlation of DHA content with viability and progressive motility of sperm cells (105).

Mendeluk et al. (106) reported that several fatty acid concentrations, including 16:1 cis9, 18:2 (ω-6, LA), 20:5 (ω-3, EPA), and 22:6 (ω-3, DHA), increased significantly in erythrocytes after dietary supplementation was provided. Recently, research efforts have explored the relationship between season and lipid profiles of bull semen (107) identified and quantified the lipid profile of semen samples from five Holstein–Friesian bulls during the summer (August to September) and winter (December to January) months. While the average volume of ejaculates and the total sperm numbers per ejaculate did not differ between seasons, sperm concentration was lower in winter than in summer. Despite lower sperm concentration in the winter months, the proportion of spermatozoa defined as morphologically normal was higher in addition to the motility, progressive motility, and velocity of spermatozoa collected in the summer months (107). Further studies could use these initial results to develop predictors of sperm fertilization competence.

### Lipidomic Techniques and Applications

The fatty acid composition of sperm cells has been a topic of investigation for several years. Previous research has elucidated groups or classes of fatty acids in spermatozoa from bulls, boars, roosters, stallions, and human males. However, quantifying the levels or amounts of these fatty acids has proven to be more difficult than qualifying the fatty acids and detecting their presence. This is a rather difficult task because of the number of cells may be limited and the calibration of the technologies used to identify and quantify the fatty acids could be set to higher threshold levels than what is present. Recent efforts have been made to design a streamlined method to fractionate then quantify the fatty acids in sperm cells via GC-MS methods (108). Lipidomics has also been utilized to identify lipid profile differences between healthy and diseased human patients. For example, blood plasma from patients with diseases, such as acute lung infections, pulmonary embolism, or acute exacerbation of the chronic pulmonary disease, had a more than 2-fold increase in various lipids compared to healthy patients (109). Lipidomics and liquid chromatography-mass spectrometry may be used to diagnose subclinical coronary artery disease (110) determined that patients with severe coronary calcification tended to have greater levels of monounsaturated triacylglycerols and saturated triacylglycerols. This led to the suggestion that calcification could be associated with cellular autophagy dysfunction.

Researchers have started to explore the possibility of using sperm as an indicator of health and risk of cancer in male subjects. For example, post-thaw semen quality of cancer patients is of lower quality as compared to samples before being frozen (111). Furthermore, men with testicular cancer have significantly lower sperm cell concentrations, but patients with other cancer types have been shown to have no differences in normal sperm (112). It has also been noted that diet affects the quality of fatty acids and stability of the sperm plasma membrane. In a study performed by Marchiani et al. (2015), rabbits were fed high-fat diets to determine if sperm quality changed due to metabolic status. The sperm cells from these rabbits showed marked decreases in motility measurement of both progressive and total motility, in addition to reduced normal morphology. The authors noted that hypertension could be a potential indicator of sperm quality in humans. These structures and their composition help determine the fertility of a given sire, but there are still many unknowns that need exploring.

A variety of microscopy tools are readily available to ascertain and evaluate the sperm membrane structure and integrity. Advances in electron microscopy has allowed for the development of a clearer, more accurate depiction of the landscape of the sperm cell. Using staining techniques in conjunction with microscopy, the composition of membrane regions has become more apparent (113). Scanning electron microscopy is commonly used to evaluate semen samples, such as in the study completed by Khalil et al. (6), which assessed the structural damage of cryopreservation by examining sperm cells for detached and cracked heads as well as damaged tails. The researchers also used transmission electron microcopy to assess the plasma membrane, acrosome, and nucleus by recording the appearance of swelling in the membrane, the typicalness of the acrosome, and the damage to the mitochondria and chromatin. In the study by Dobranić et al. (114), functional membrane integrity of canine spermatozoa was evaluated using hyper-osmotic swelling test (HOST). With HOST, sperm cells are incubated in a hypoosmotic solution such as fructose solution with Na-citrate to determine intactness of membranes in the sperm cells. Sperm with curled or more flaccid tail appearance indicate intact or damaged tails, respectively (115).

Lipidomics involve characterization of lipid content and their biological roles in each biological sample using analytical methods. Currently, there are two strategies for the lipid analyses: targeted and non-targeted lipid analysis. Targeted lipidomics is applied when researchers focus on known and specific lipids. Since the selected reaction monitoring (SRM) method is utilized in targeted lipidomics, it provides high sensitivity for quantitative lipid analyses 44, 80 [(116); 101]. Lipid classes that show unique fragmentation patterns and low abundant lipids are suitable for targeted lipid analyses. Non-targeted lipid analysis helps detect all lipids simultaneously in a single run. Although this method provides an overall profile of lipids that are detectable, it is not a sensitive analysis. Combining targeted and non-targeted lipid strategies may help to produce more powerful data.

Since lipidomic techniques are relatively new, several challenges exist. The use of gas chromatography-mass spectrometry (GC-MS) to elucidate lipid profiles has proven to be a promising avenue for determination of bull fertility, but this machinery and use of the technology are not widely available and it requires trained personnel to produce reliable data. One of the major limitations often encountered is the lack of subjects or samples utilized for analysis. Having a greater number of cells to analyze could provide more comprehensive results or lead to the discovery of other compounds. In our recent study, a GC-MS method was used to evaluate the differences in Holstein bull sperm freezability and to compare the quality and quantity of fatty acids (108). When compared to similar studies, we noted that the calibration and detection techniques can vary which will yield different results, thereby making the use of GC-MS beneficial because you can collect a breadth of spectral data while also challenging due to the number of variables and settings that can alter specificity of the analysis.

With gas chromatography, modifications and adjustments can be made to the gas flow rate, column specifications, and temperature which can prove beneficial when quantifying lipids. Gas chromatography is an analytical tool that allows for the separation of compounds via vaporization. The carrier gas transports the injected liquid sample. Carrier gases are typically inert gases, such as helium. The sample is carried from the injector to the column that is located within the oven (117). Columns vary in length, ranging from a couple of meters to 100 meters, and type, such as polarized vs. non-polarized. Common detectors used with GC are MS or flame ionization (FID). Compounds assessed using GC should be compared against a standard for validation (118). Internal standards can be obtained for the various lipids, but not with the same ease of access. Approximately 80 analytical standards are commercially available for GP of the complex category, limiting the ability to perform absolute quantification (118). Sample preparation is another drawback of GC in that it typically requires using large sample volumes in addition to samples requiring derivatization (119).

Much like GC, a standard is needed for absolute quantification, and for simple lipids these are available commercially. Mass spectrometry (MS) is another analytical technique. By using the masses of atoms and molecules, the identities of the various components that make up a sample is revealed. The data gathered can also be used to quantify the components of the sample as well. The MS works by converting molecules to ions, sorting the ions based on their mass and charge, and then, detection. The electron ionizer is an electron beam that molecules pass through that strips the electrons, thus, producing a positive ion that travels to the mass analyzer component, which is an electric field that accelerates the ions into a magnetic field where they are then deflected based on the mass of the ions. Lastly, the ions impact a charged plate that generates a signal that can be used for analysis (120). The MS is useful in quantifying a substance when it is known and determining the composition of an unknown sample, in addition to, allowing researchers to conclude the structure and properties of various molecules (118). The MS determines the abundance of ions according to their mass to charge ration or m/z (119). When compared to nuclear magnetic resonance (NMR), MS often offers heightened sensitivity and selectivity between various lipids (121).

Recent advances in analytical technologies, such as MS, NMR, and high-performance liquid chromatography (HPLC), have helped researchers to improve lipidomics (121). Among these technologies, MS-based methods are commonly used in lipid analyses due to the higher sensitivity, throughput, and specificity (122). In addition, a great number of ionization technologies, such as electron ionization (EI), Matrix-Assisted Laser Desorption Ionization (MALDI), Electrospray Ionization (ESI), and Fast Atom Bombardment (FAB) in MS, have been developed as well. Each of these ionization methods can be used for the analyses of different lipid groups, such as FAB commonly being applied to identify fatty acids, monoacylglycerols, and glycerophospholipids (123, 124).

Although the NMR is not as sensitive as MS, NMR is the only method of analysis that allows for lipid analysis of cells and tissues when they are intact (119). Nuclear magnetic resonance spectroscopy is composed of a coiled wire surrounded by a magnet. One of the coils generates electromagnetic radiation at a constant frequency, whereas the relative strength of the magnetic field increases. The growing magnetic field strength splits the nuclei in the samples until the nuclei reach a point of resonance, after which, the nuclei fall back to a lower energy level remitting a radiation signal that the second coil records. The signals recorded by the various nuclei in the sample are then analyzed and processed producing the NMR spectrum (125). Typically, 1H and 31P NMR spectroscopy are used for analysis due to their sensitivity. Proton NMR is commonly used to investigate diseases, poisons, and disorders that induce changes in the lipid composition; 31P NMR is commonly used to quantify GPs. In the past, one-dimensional NMR has been the most prevalent tool, however, two-dimensional NMR is becoming a useful tool. The rise in popularity for two-dimensional NMR is centered around the ability of better resolution (118).

*The thiobarbituric acid (TBARS) assay* is used to assess changes in Malonaldehyde (MDA), a reactive compound formed when lipids undergo oxidation (126). In conjunction with Thiobarbituric acid (TBA), MDA reacts to form the MDA-TBA adduct and can be measured colorimetrically or fluorometrically to determine the levels of lipid peroxidation in each sample (126). The TBARS assay needs to be carried out under high temperatures and in an acidic environment. To run this assay, semen samples are thawed and diluted in PBS (127). Then, 100 μL of spermatozoa are mixed with 200 μL of 5% trichloroacetic acid, 0.375% TBA and 0.25 N HCl reagent. The mixture is then heated to 90°C for 15 min to stimulate the reaction. Following the incubation period, samples are transferred to an ice-water bath for 5 min. After cooling, the samples are centrifuged at 1,500 × g for 15 min. The supernatant is then collected and transferred into a well-plate so the absorbance can be measured by a microplate reader to calculate MDA concentration. This method has the benefit of being well-recognized and can utilize a variety of sample types such as tissue homogenates, urine samples, cell lysates, serum, and plasma. However, it is necessary to standardize TBARS by using multiple fatty acid concentrations rather than selecting an arbitrary fatty acid to use as a standard or reference (128). This method lacks specificity, but it can help determine the amount of lipid peroxidation present if the sample is uncomplicated (127, 128).

*The BODIPY C*_11_ *probe colorimetric assay* measures lipid peroxidation of cell membranes via flow cytometry. BODIPY (581/591) C_11_ easily incorporates into sperm cells and undergoes a spectral emission shift when attacked by ROS that can be measured to determine change (129). To conduct this assay, semen samples are collected, and then, allowed to sit for 30 min to liquify from its gel-like stage post-ejaculation (129). Following the waiting period, sperm cells are separated via a Percoll gradient, and then, the BODIPY (581/591) C_11_ probe is added to 5μM of cells for 30 min. Sperm cells are washed twice by centrifuging at 650 g for 5 min. An 80 μM ferrous sulphate promoter is incubated for 15 min. The sample is then evaluated using a flow cytometer. The BODIPY probe colorimetric assay has been demonstrated to have good repeatability and sensitivity when evaluating deer sperm (127).

*The TBA-TCA Reagent Colorimetric Method Assay* is used to measure lipid peroxidation by determining MDA levels through the TBA assay, which produces a red absorbance. This assay is run by thawing and centrifuging sperm cells in Tris buffer (130). The sperm pellet is then resuspended in PBS. A 2 mL of TBA-TCA reagent is added to 1 mL of sperm cell suspension and incubated in boiling water for 40 min. The sample is cooled and centrifuged at 500 × g for 10 min. The supernatant is aspirated, and absorbance is read at 535 nm under a UV spectrophotometer. Final MDA levels are determined by the absorbance coefficient of 1.56 × 105/mol/cm^3^. The TBA can react with a wide assortment of oxidized lipids, both saturated and unsaturated varieties, but it does lack sensitivity and specificity (131). To combat these weaknesses, researchers have incorporated high-performance liquid chromatography to increase specificity and sensitivity of the assay (132).

*The 4-Hydroxynonenal (HNE)-His Adduct ELISA/HNE Adduct Competitive ELISA* is an immunoassay that helps detect HNE-His protein adducts, which are formed when 4-HNE reacts with lysine, histidine, or cysteine residues in sperm cells (133). This assay is run with a 96-well titer ELISA plate where sperm cell samples and bovine serum albumin (BSA) standards are added to wells (134). The HNE-protein adducts present in the samples are probed with an anti-HNE-His antibody, followed by an HRP secondary antibody. Using a microplate reader, the absorbance of each well is read at 450 nm to quantify the HNE-protein adducts. This method has proven to be accurate and repeatable; however, care must be taken when selecting antibodies for the sample specimen (133, 135).

In the *Glutathione peroxidase test*, glutathione peroxidase (GSH) reacts with hydrogen peroxide to form glutathione disulfide (GSSG). Adding glutathione reductase and NADPH reduces GSSG to GSH and results in consumption of NADPH, which is related to the peroxide content of the sample (136). Sperm cell samples are centrifuged at 12,000 g for 5 min (137). Fifty μL of sperm cells are added to a 930 μL solution of EDTA 1 mM, sodium azide, and potassium phosphate buffer (137). Then, a 10 μL secondary solution, composed of 0.02 g of 1-chloro-2,4-dinitrobenzene (CDNB) in ethanol, is placed into the cuvette of the spectrophotometer with the aliquots of the first solution. Finally, 20 μL of 500 IU/mL of GSH-S transferase in phosphate buffer is added to initiate reaction (137). The absorbance is monitored at 340 nm until it reached the plateau. Calculations are then performed using the volume of the sample, light path length, corresponding dilution factors, absorbance decrease, and molar extinction coefficient. This test has been applied to human seminal plasma samples to quantify the presence of glutathione peroxidase (138). It was found that glutathione peroxidase activity was significantly lower in those samples with oligozoospermia, asthenozoospermia, or teratozoospermia conditions in which Crisol et al. (138) speculate is related to overall sperm quality. When utilizing this test, it is vital to consider other avenues of assessment because this test only evaluates one fraction of the antioxidant system that is in place to protect the spermatozoa.

## Conclusions

The knowledge base of lipids and their composition in livestock sperm and the difficulty of data accuracy and interpretation of results have been documented. There is a need for more detailed lipidomics studies utilizing sperm from livestock with distinct phenotypes of economically important traits such as sperm freezability and male fertility. Growing interests and platforms with various techniques such as GC-MS, MS-MS, and LC-MS enable researchers to profile comprehensive metabolomic signatures of diverse tissues in livestock, including sperm. This is important because lipids play critical roles in molecular morphology and function in the cells. Combined with other methods in cell and molecular biology, such as bioinformatics, lipidomics can be applied to harness the power of integrated studies to decipher sperm markers for freezability and male fertility. Potential markers uncovered through discovery research can be further studied through mechanistic experiments to determine the molecular and cellular underpinnings of male fertility. However, there is a need for more comprehensive studies involving different stages of animal development, nutrition, environment, and season using single cell analyses. Because of the significant similarities between livestock and other organisms, including human and endangered species, results generated using various livestock models can be applied to advance basic and applied reproduction of other mammals.

## Author Contributions

HE, TD, MLH, AG, MU, MH, FJ, MN, and EM assisted in the conception of the study and contributed to manuscript revision, read, and approved the submitted version. All authors contributed to the article and approved the submitted version.

## Conflict of Interest

The authors declare that the research was conducted in the absence of any commercial or financial relationships that could be construed as a potential conflict of interest.
